# Genomic analysis of cellular hierarchy in acute myeloid leukemia using ultrasensitive LC-FACSeq

**DOI:** 10.1038/s41375-021-01295-1

**Published:** 2021-05-21

**Authors:** Caner Saygin, Eileen Hu, Pu Zhang, Steven Sher, Arletta Lozanski, Tzyy-Jye Doong, Deedra Nicolet, Shelley Orwick, Jadwiga Labanowska, Jordan N. Skinner, Casey Cempre, Tierney Kauffman, Virginia M. Goettl, Nyla A. Heerema, Lynne Abruzzo, Cecelia Miller, Rosa Lapalombella, Gregory Behbehani, Alice S. Mims, Karilyn Larkin, Nicole Grieselhuber, Alison Walker, Bhavana Bhatnagar, Clara D. Bloomfield, John C. Byrd, Gerard Lozanski, James S. Blachly

**Affiliations:** 1 Department of Internal Medicine, The Ohio State University, Columbus, OH USA; 2 Division of Hematology, The Ohio State University Comprehensive Cancer Center, Columbus, OH USA; 3 Alliance Statistics and Data Center, The Ohio State University, Columbus, OH USA; 4 Department of Pathology, The Ohio State University, Columbus, OH USA

**Keywords:** Acute myeloid leukaemia, Cancer genetics

## Abstract

Hematopoiesis is hierarchical, and it has been postulated that acute myeloid leukemia (AML) is organized similarly with leukemia stem cells (LSCs) residing at the apex. Limited cells acquired by fluorescence activated cell sorting in tandem with targeted amplicon-based sequencing (LC-FACSeq) enables identification of mutations in small subpopulations of cells, such as LSCs. Leveraging this, we studied clonal compositions of immunophenotypically-defined compartments in AML through genomic and functional analyses at diagnosis, remission and relapse in 88 AML patients. Mutations involving DNA methylation pathways, transcription factors and spliceosomal machinery did not differ across compartments, while signaling pathway mutations were less frequent in putative LSCs. We also provide insights into *TP53*-mutated AML by demonstrating stepwise acquisition of mutations beginning from the preleukemic hematopoietic stem cell stage. In 10 analyzed cases, acquisition of additional mutations and del(17p) led to genetic and functional heterogeneity within the LSC pool with subclones harboring varying degrees of clonogenic potential. Finally, we use LC-FACSeq to track clonal evolution in serial samples, which can also be a powerful tool to direct targeted therapy against measurable residual disease. Therefore, studying clinically significant small subpopulations of cells can improve our understanding of AML biology and offers advantages over bulk sequencing to monitor the evolution of disease.

## Introduction

Acute myeloid leukemia (AML) is an aggressive malignancy, characterized by clonal expansion of myeloid progenitors, with impaired normal hematopoiesis. Our understanding of clinical and biological heterogeneity of AML has improved substantially over the past two decades [1–4]. Next-generation sequencing (NGS) has been used to identify recurrent myeloid mutations and become part of routine clinical evaluation for a patient with newly diagnosed AML [5]. Information obtained from DNA sequencing of the bulk of the tumor (e.g., whole blood or marrow) is important for prognostication and recently, efforts have also demonstrated its importance in treatment selection [6]. Younger and fit elderly patients often receive induction chemotherapy, followed by consolidation treatment with more chemotherapy or hematopoietic cell transplantation (HCT). Novel targeted therapies approved within the past 4 years improved outcomes in AML patients with select genomic aberrations. Despite these advances, most patients relapse and die of AML. Genetic heterogeneity of precursor hematopoietic stem cells throughout the clonal evolution of AML is not completely understood and has been limited in part by the inability to study genomic DNA abnormalities in very small cell numbers.

Normal hematopoiesis is organized in a hierarchical manner and it has been hypothesized that AML is organized in a similar way with leukemia stem cells (LSCs) at the top of hierarchy, giving rise to more differentiated blasts to sustain AML [7, 8]. The functional in vivo evidence of such organization is the enhanced colony formation and serial engraftment ability of a small fraction of leukemic cells expressing hematopoietic stem cell (HSC) markers (e.g., CD34^+^CD38^–^) [9, 10]. In most AML patients, LSCs can at least partially be defined by cell surface markers or functional assays. LSCs represent a very minor fraction of bulk leukemia and harbor unique characteristics that account for increased self-renewal, chemoresistance and quiescence [7]. The frequency of LSCs at diagnosis may inform prognosis, as evidenced by the inferior survival outcomes of AML patients with high CD34^+^CD38^–^ cell frequency [11, 12]. LSCs can also evolve throughout the disease course. At relapse, persistent LSC clones from diagnosis may emerge as dominant clones, or preleukemic founder clones may acquire new mutations and re-emerge with new subclonal compositions [13]. Therefore, deployment of new methods of monitoring may provide an opportunity to implement new therapeutic approaches targeting this minority of cells to prevent relapse.

Most hematopoietic cells are short-lived, thus the accumulation of multiple mutations in a single clonal lineage suggests a stepwise acquisition starting at self-renewing HSCs [14]. More mature non-self-renewing cells may acquire a mutation, but this would be lost unless the mutation confers self-renewal ability. Similar to most cancers, the incidence of AML increases with age. A precursor condition, named age-related clonal hematopoiesis (CH), was described after identification of somatic mutations in HSCs and mature blood cells of elderly persons with no hematologic compromise [15, 16]. The most common CH-associated mutations involved *DNMT3A, TET2*, and *ASXL1* (DTA) genes, and individuals with these had much higher risk of developing myeloid malignancies, including AML. Therefore, myeloid cells in the CH compartment may have a survival advantage over their normal counterparts and can also accumulate additional mutations, thus becoming LSCs. There is considerable effort in identifying persons who should be screened for CH, with the goal of developing preventative strategies against myeloid cancers [17, 18]. However, patterns of stepwise mutation acquisition from self-renewing CH stem cells to LSCs, and subsequently to non-self-renewing proliferative cells of overt leukemia (non-LSCs) should be established to guide our preventative efforts in clinic.

The low frequency of cells residing in the LSC compartment represents a challenge for detection and genomic characterization. Similarly, a small and persistent population of cells after treatment, termed as measurable residual disease (MRD), is often enriched with LSCs but can be difficult to monitor. The ability to do this is important as specific targeted therapy might be applied to MRD that emerges from therapy and did not pre-exist. Current clinical DNA sequencing and MRD monitoring techniques (e.g., flow cytometry, NGS) use bulk tumor cells and lack the ability to easily track clonal evolution in rare tumor cells [19]. We have recently developed a highly sensitive technique for molecular characterization of rare leukemic cells in the blood and bone marrow [20]. Limited cells acquired by fluorescence activated cell sorting in tandem with targeted amplicon-based sequencing (LC-FACSeq) technique enables accurate identification of mutations in immunophenotypically defined subpopulations of cells. This technique has been validated in both AML and chronic lymphocytic leukemia (CLL) [20]. Leveraging this novel method, we investigated the clonal composition of self-renewing and non-self-renewing cell populations in AML through genomic and functional analyses at diagnosis, remission and relapse.

## Materials and methods

### Patients and samples

Bone marrow, leukapheresis and blood samples of AML patients were obtained with informed consent under protocols approved by The Ohio State University Comprehensive Cancer Center (OSUCCC) Institutional Review Board. Samples at diagnosis were obtained before initiation of therapy. We excluded CD34^–^ AML cases, defined as <5% positivity on leukemic blasts. Mononuclear cells were isolated using Ficoll density gradient centrifugation within 24 h of collection. All analyses conducted here used freshly thawed cells or fresh primary human samples as available.

For clinical correlative analyses, cytogenetic risk was ascribed per European LeukemiaNet (ELN) 2017 guidelines [5]. Karyotyping was performed in clinical laboratory as part of routine patient care and data were retrieved from medical records. Complete remission (CR) was defined as the bone marrow blast <5%, absence of circulating blasts or extramedullary disease, absolute neutrophil count >1000/µL and platelet count >100,000/µL. CR with incomplete count recovery (CRi) meets all CR criteria except count recovery [5].

### Colony formation assays

For colony formation assays, AML cells were counted and plated in ~1000 cells per well in 12-well culture plates. After 14 days of incubation in MethoCult H4034 Optimum media (Stemcell Technologies, Vancouver, BC, Canada), individual colonies were counted and isolated for sequencing experiments. For the replating experiments, cells were collected from the MethoCult medium, washed, counted and replated at the same densities. Colonies were counted after an additional 14 days. To assess self-renewal frequencies of the cells residing in different immunophenotypic compartments, we used Extreme Limiting Dilution Analysis (ELDA) platform. Namely, cells were sorted in duplicate rows of serial dilutions into 96-well ultra-low-attachment plates with 100 μL Methocult H4034 Optimum media per well. Colonies were counted at the end of 14 days and stem cell frequencies were calculated by using the ELDA platform (http://bioinf.wehi.edu.au/software/elda/) [21].

### Flow cytometry and cell sorting

Human AML cells at a concentration of 1 million cells/mL were sorted on BD FACS Aria III to isolate immunophenotypically defined HSC (CD45^dim^SSc^low^Lin^–^CD90^+^CD34^+^CD38^–^), LSC-enriched (CD45^dim^SSc^low^ Lin^–^CD90^–^CD34^+^CD38^–^), and non-LSC-enriched (CD45^dim^SSc^low^Lin^–^CD90^–^CD34^+^CD38^+^) populations. The anti-human antibodies used for FACS analysis were FITC-CD34 (clone 561, Biolegend), PE-CD38 (clone HIT2, Biolegend), APC-CD45 (clone HI30, Biolegend), Pacific Blue-Lineage cocktail (#348805, Biolegend) combined with Pacific blue-CD138 (clone MI15, Biolegend) to exclude plasma cells, APC/Cy7-CD90 (clone 5E10, Biolegend), BV421-CD99 (clone 3B2/TA8, Biolegend), Pacific Blue-CD123 (clone 6H6, Biolegend), and APC-TIM3 (clone F38-2E2, Biolegend). Briefly, cryopreserved or fresh mononuclear cells from AML patients were stained with antibody cocktail on ice for 30 min before being washed once and resuspended in sorting buffer. A high purity single cell sorting strategy was used for cell numbers less than 500 into PCR strip tubes. Bulk leukemic blasts (>20,000 cells) were sorted into 1.5 mL Eppendorf tubes. Purity of sorting achieved for all samples were >98%.

### Genomic DNA preparation

For bulk leukemic blasts with cell number yield >20,000 cells, gDNA was extracted using the Puregene Core kit A DNA extraction kit (Qiagen, Maryland, USA) according to manufacturer instructions. After quantification, 10 ng of gDNA was used for amplification step.

For sorted HSC, LSC, non-LSC compartments with cell number yield of 300–500 cells and single colonies collected from methylcellulose medium, cells were lysed immediately post-collection by using the Single Cell Lysis Kit (Invitrogen) as described before [20]. Briefly, 9 µL of single cell lysis solution was directly added to the droplet of sorted cells or collected colony. After 5 min incubation at room temperature, 1 µL of stop solution was added. The reaction mixture was used for amplification.

### Targeted sequencing with Ion Torrent AmpliSeq

Sequencing method of LC-FACSeq was described previously for both CLL and AML [20]. To summarize, libraries were prepared by amplifying gDNA using Ion AmpliSeq Library Kit 2.0 with a custom designed panel of AmpliSeq primers (IAD145356, 129 amplicons, 2 pools, 15.32 kb panel size, coverage 100%) **(**Supplementary Table 1**)**. PCR products were barcoded with IonExpress barcode adapters, and barcoded products were purified with Agencourt AMPure XP kit (A63881 Beckman Coulter, Indianapolis, IN). Libraries constructed with “extracted DNA” from bulk leukemic blasts were directly quantified at this point, while libraries constructed from 300–500 cells underwent an additional amplification cycle to equalize the library amounts. Upon quantification with Ion Library TAQMAN Quantitation kit, diluted libraries were loaded onto the OneTouch OT2 version instrument. ISP enrichment and purification were performed on Ion One Touch2 ES and purified ISPs were analyzed on the Ion Torrent personal genome machine using the Ion PGM Hi-Q sequencing kit (A25591). ISPs were also analyzed with the Ion Torrent S5 instrument using the Ion 530/540 Chip Kit (Life Technologies, Carlsbad, CA). Data were analyzed using the Torrent Server with Torrent Suite 5.0 version. A combination of the following software was used for final analysis of sequencing data: Variant Caller v.5.0.28 and v.5.6.8-1, IGV v5.01, Ion Reporter v.4.6, Samtools v.1.6, Bcftools v.1.31 and Ensembl Varian Effect Predictor v.92.03. The hg19 reference was used for analysis. Lastly, we manually reviewed the entire length of sequences using these programs to assess for deviation from reference sequence and to evaluate the quality of sequence and depth of coverage. For germline confirmation, mutations and their frequencies were compared between different compartments as well as CD3+ T cells whenever DNA was available. The mutant allele frequency was determined as mutant read number/(germline read number + mutant read number).

### Fluorescence in situ hybridization (FISH)

At least 10,000 FACS-purified cells from immunophenotypically defined subpopulations in *TP53* mutated AML patients were subjected to FISH. Namely, cells were pelleted and fixed with Carnoy’s fixative (Methanol and Glacial Acetic Acid at 3:1 ratio) immediately after the sorting. *TP53*(17p13.1)/ATM(11q22.3) FISH probes (Abbott Molecular, Des Plaines, IL) were used. Hybridization was performed according to the manufacturer’s directions. One hundred cells were analyzed in each fraction.

### Animal studies

Nonobese diabetic/severe combined immunodeficiency mice with an interleukin-2 receptor gamma chain mutation (NSG) were purchased from The Jackson Laboratory (Bar Harbor, ME). Mice were 4–6 weeks old at experiment initiation. Experiments were approved by The OSU Institutional Animal Care and Use Committee. Leukemia stem cells obtained from the bone marrow of AML patients were engrafted into NSG mice via tail vein injection (10,000 cells per mouse), and monitored for development of leukemia. Mice meeting early removal criteria (ERC) were sacrificed and their bone marrow and spleen were harvested for further studies. The researchers were not blinded to groups.

### Statistical analyses

Clinical data are presented with percentage proportions for categorical variables and medians for continuous variables. The chi-square test and Fischer’s exact test were used to compare categorical variables. Comparisons for two groups of continuous variables were made with two-tailed Student’s *t* test. Comparisons between >2 groups of continuous variables were made with one-way ANOVA. Survival estimates were calculated using the Kaplan–Meier method and differences between curves were assessed using the log-rank test. The overall survival (OS) time was calculated from the time of diagnosis until death or last follow-up. Relapse-free survival (RFS) was calculated from the time point of CR/CRi until the time of relapse, death or the last follow-up. Patients were censored at the time of HCT for these analyses. Patients who did not experience these events were censored at last follow-up. Statistical analyses were performed using JMP software v.14.0.0 (SAS Inc, Cary, NC) and *p* ≤ 0.05 was considered statistically significant. Graphs were created using GraphPad Prism version 7 (GraphPad Software, La Jolla, CA). For clonal structure inference, mutations belonging to the largest clone were defined as dominant mutation and other clones as secondary mutations. Fishplots visualizing the clonal evolution were generated with R statistical software.

## Results

We have previously reported the validation of LC-FACSeq method with low cell numbers in AML [20]. With an average sequencing depth of 1212 (SEM = 56) per gene and average coverage uniformity of 88.24% (SEM = 0.01%), we created a Poisson/β-binomial model to assess any deviation of VAF by using low cell numbers. When DNA-isolated and purified bulk population (>20,000 cells) was compared to low-number cell lysates in AML samples, the median deviation from true VAF was −0.2% (interquartile range, −3.5 to 0.1) for 300 cells, and 0% (interquartile range, −2.6 to 0.1) for 500 cells [20]. Therefore, LC-FACSeq enables us to interrogate genomic heterogeneity in low number cell populations. In this present report, we included a bulk population with >20,000 cells as a comparative group to sort-purified fractions.

### LSCs can be isolated with LC-FACSeq technology

**Fig. 1:**
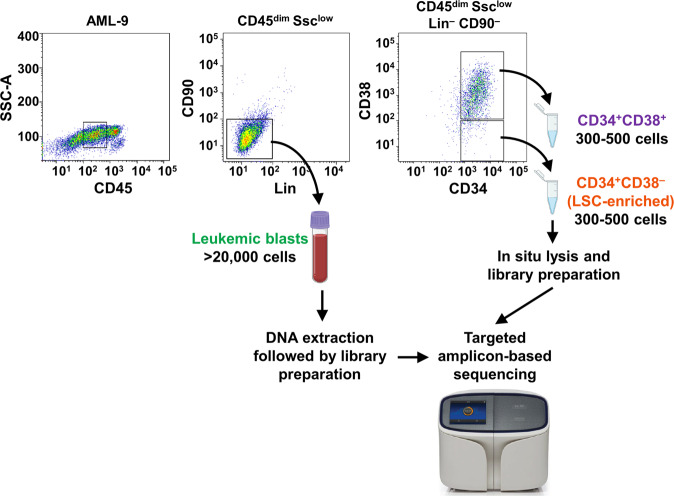
Isolation and sequencing of immunophenotypic leukemia stem- and non-stem cell compartments from primary acute myeloid leukemia (AML) samples by using LC-FACSeq. A multicolor flow cytometry panel was used to enrich the subpopulation of cells with high self-renewal frequency, defined as the immunophenotypic (CD45^dim^SSc^low^Lin^–^CD90^–^CD34^+^CD38^–^) leukemia stem cell (LSC) compartment. Immunophenotypic LSCs and their non-LSC counterparts were subjected to immediate lysis upon enrichment, followed by preparation of libraries for next generation sequencing. Bulk leukemic blast population (CD45^dim^SSc^low^Lin^–^CD90^–^) was also sorted, but subjected to traditional DNA extraction protocol, followed by library preparation. All samples were sequenced with a targeted amplicon-based sequencing platform.

To obtain direct insights into the hierarchical organization and molecular heterogeneity of tumor cells in AML, we studied 88 treatment-naïve AML patients diagnosed between 2003 and 2018 at OSUCCC with pre-treatment blood, leukapheresis or bone marrow sample obtained at diagnosis. Clinical characteristics of these patients are summarized in Table 1. For all patients, we used multiparameter FACS to fractionate phenotypically defined bulk leukemic blasts (CD45^dim^SSc^low^ Lin^–^CD90^–^), LSC-enriched (CD45^dim^SSc^low^ Lin^–^CD90^–^CD34^+^CD38^–^) and non-LSC-enriched (CD45^dim^SSc^low^ Lin^–^CD90^–^CD34^+^CD38^+^) (Fig. 1). The bulk blast population contained >20,000 cells, from which DNA was extracted to compare with low cell number LSC and non-LSC populations which were subjected to in situ lysis followed by library preparation. All populations were sequenced with targeted AmpliSeq panel as described.

**Table 1:** Clinical characteristics of acute myeloid leukemia (AML) patients (*n* = 88).

Patient characteristics	
Age, median, yrs (range)	60.5 (22–87)
Female, *n* (%)	31 (38)
AML type, *n* (%)	
De novo	57 (65)
Secondary	20 (23)
Therapy-related	7 (8)
Unknown	4 (4)
ELN 2017 risk, n (%)	
Favorable	19 (22)
Intermediate	38 (43)
Adverse	31 (35)
WBC count (per µL), median (range)	45 (0.5–409)
Peripheral blast %, median (range)	47 (0–98)
Bone marrow blast %, median (range)	63.5 (9–99)
Initial therapy, *n* (%)	
7 + 3 based	52 (59)
HMA	12 (14)
Palliative	10 (11)
Unknown	14 (16)
Response to initial therapy, *n* (%)	
CR/CRi	40 (45)
Refractory	28 (32)
Unknown	20 (23)
Performance of HCT, *n* (%)	
In CR1	14 (16)
In CR2	8 (9)
Without remission	2 (2)
No HCT	57 (65)
Unknown	7 (8)
Median OS, months (range)	14 (0.5–183)
Median RFS, months (range)	12 (1–182)

*CR* complete remission, *CRi* CR with incomplete count recovery, *ELN* European Leukemia Net, *HCT* hematopoietic cell transplant, *HMA* hypomethylating agent, *OS* overall survival, *RFS* relapse-free survival, *WBC* white blood cell; 7 + 3, 7-days of cytarabine and 3-days of daunorubicin.

Similar to other reports [11, 12], LSC-enriched compartment represents a minor subpopulation of leukemic cells with a median frequency of 0.5% (range, 0.01–69%) (Supplementary Fig. 1A). We further validated our immunophenotypic markers in human AML cells by comparing self-renewal frequencies of sorted compartments (Supplementary Fig. 1B). In vitro limiting dilution analysis showed that CD34^+^CD38^–^ compartment was significantly enriched with self-renewing LSCs when compared to bulk leukemic blast population and CD34^+^CD38^+^ population. Therefore, AML LSCs represent a minor fraction of blasts at diagnosis and can be enriched in CD34^+^CD38^–^ compartment with multicolor FACS.

Available samples used for the analyses included bone marrow specimens for 42 patients (48%), apheresis specimen for 29 patients (33%), and peripheral blood for 17 patients (19.3%). Although immunophenotypic selection of compartments would eliminate mature blood cell contamination, we performed a comparative analysis of VAFs for variants detected in apheresis vs bone marrow sample from the same patient (Supplementary Fig. 1C). There were no significant differences between sample sites for all compartments.

### LSC frequency at diagnosis can predict patient outcomes

**Fig. 2:**
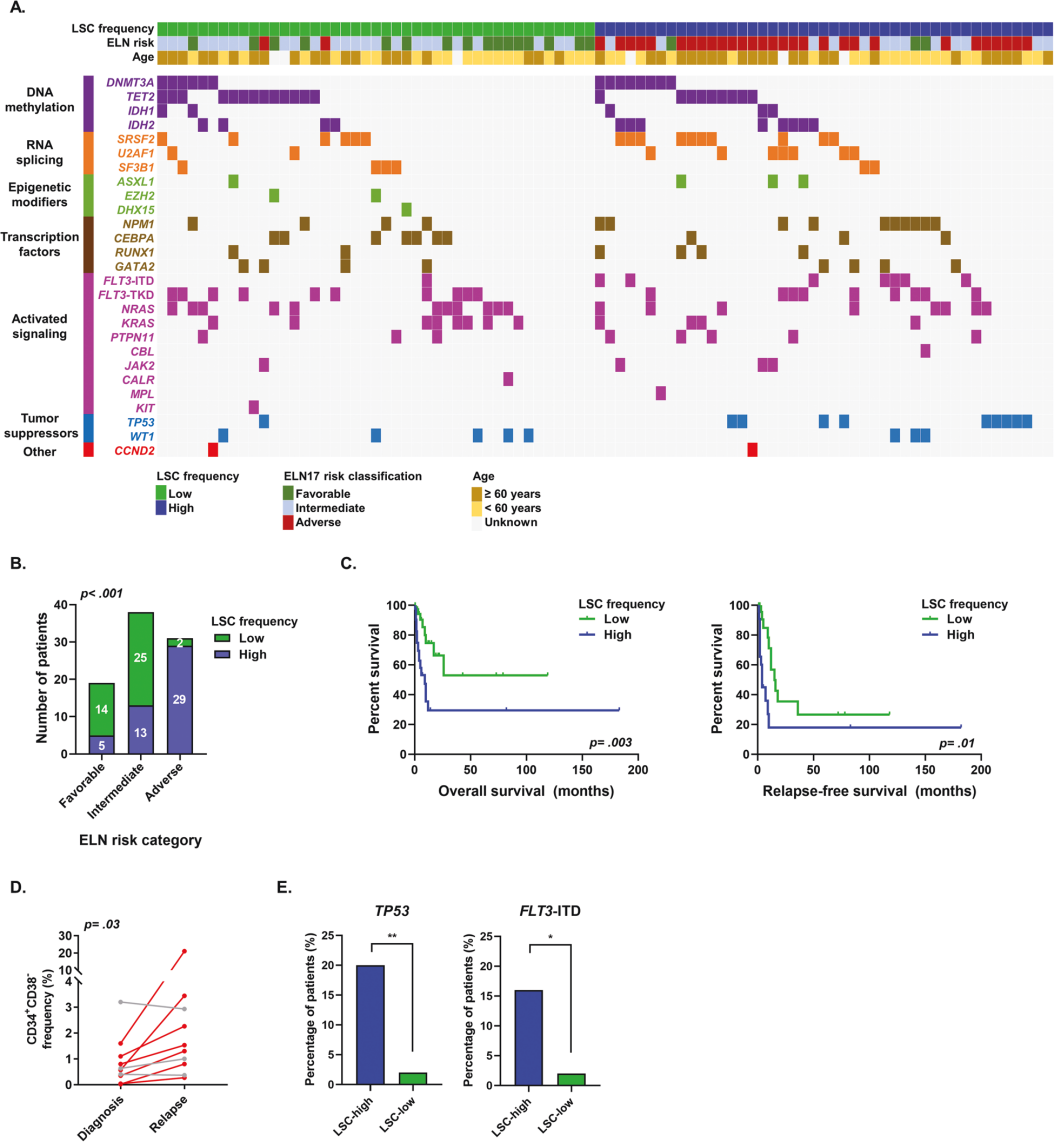
Immunophenotypic leukemia stem cell (LSC) frequency impacts on outcomes of patients with acute myeloid leukemia (AML). **A** Oncoprint of commonly occurring myeloid mutations in 88 AML patients. **B** Bar graph demonstrating distribution of AML patients stratified based on European Leukemia Net (ELN) prognostic risk groups and LSC frequency. The cutoff for high vs low LSC frequency was 0.5% (the median value for entire cohort). **C** Kaplan–Meier overall and relapse-free survival curves of AML patients stratified based on high (≥0.5%) vs low (<0.5%) LSC frequency at diagnosis. **D** The frequency of immunophenotypic LSCs (CD45^dim^SSc^low^Lin^–^CD90^–^CD34^+^CD38^–^) in consecutive diagnosis and relapse samples of 10 AML patients. Gray lines indicate no statistically significant change, while red lines indicate a significant difference between the two VAFs. **E** *TP53* and *FLT3*-ITD mutations were significantly more common among patients with high LSC frequency as compared to patients with low LSC frequency at diagnosis. *p < 0.05, **p < 0.01.

We analyzed myeloid mutations in our AML cohort stratified based on immunophenotypically-defined LSC frequency and ELN17 risk groups [5] (Fig. 2A). Since the median LSC frequency of this cohort was 0.5%, we defined high and low LSC burden based on this cutoff. Similar to previous reports, the prevalence of high LSC frequency (≥0.5%) was significantly higher in patients with adverse risk AML defined by the ELN criteria, as compared to intermediate and favorable risk groups (94% vs 34% vs 26%, respectively, *p* < 0.001) (Fig. 2B). When compared to patients with low LSC frequency (<0.5%), those with high LSC frequency had worse OS (median, not reached vs 9 months, *p* = 0.003) and RFS (median, 15 vs 4 months, *p* = 0.01) (Fig. 2C). In addition, we did paired analysis of diagnosis and relapse samples for 10 patients, 7 of whom had higher LSC frequencies at the time of relapse (*p* = 0.03) (Fig. 2D, Supplementary Fig. 1D). However, in multivariable analysis, LSC frequency did not retain its significance after adjusting for ELN risk stratification, suggesting that it is not an independent predictor.

The most common mutations in this cohort involved *FLT3* (28%), *TET2* (25%), *NRAS* (22%), *SRSF2* (19%), *DNMT3A* (17%), *IDH1/2* (16%), and *NPM1* (16%) genes. Patients who presented with high LSC burden (≥0.5%) at diagnosis had higher incidence of *TP53* (20% vs 2%, *p* = 0.009) and *FLT3*-ITD (16% vs 2%, *p* = 0.03) mutations when compared to patients with low LSC frequency (Fig. 2E). There were no significant differences for the distribution of other mutations between these groups. Collectively, these results indicate that high LSC frequency at presentation is associated with ELN adverse-risk cytogenetic and mutational profiles, and poorer OS and RFS.

### Mutations involving signaling pathways are enriched in non-LSC compartment

**Fig. 3:**
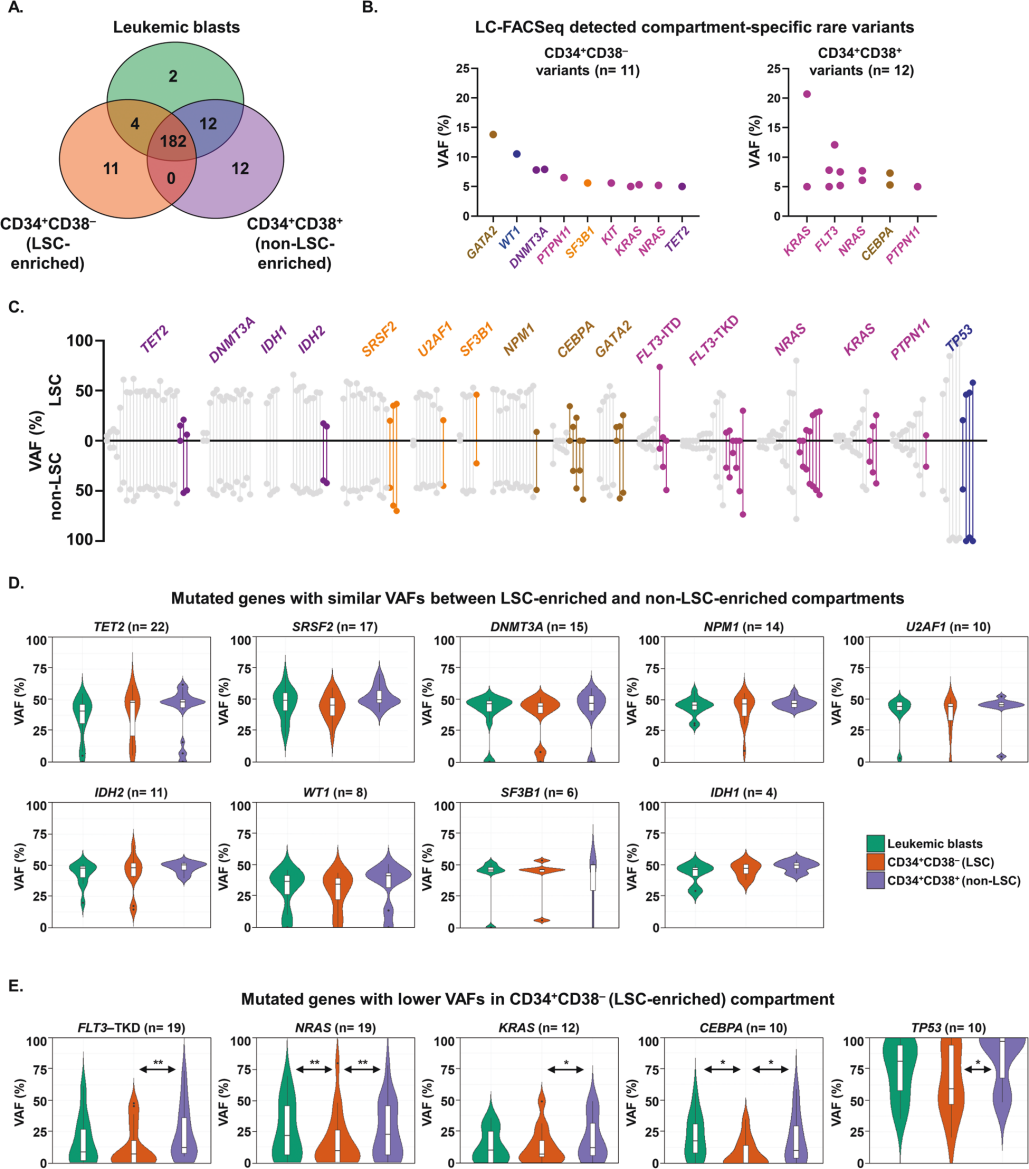
Myeloid mutations have different distributions between acute myeloid leukemia stem- and non-stem cell compartments. **A** Venn diagram showing the distribution of all pathogenic somatic sequencing variants detected by 27-gene targeted sequencing panel in immunophenotypic leukemia stem cell (LSC), non-LSC and bulk leukemic blast populations. **B** Graphs demonstrate variant allelic frequencies (VAF) of rare pathogenic variants that were observed exclusively in LSC and non-LSC compartments. **C** Lollipop plot comparing VAFs of mutations between immunophenotypic LSC and non-LSC compartments. Gray lines indicate no statistically significant difference, while colored lines indicate a significant difference between the two VAFs. **D, E** Violin plots demonstrate comparisons of VAFs between leukemic blasts, immunophenotypic LSCs and non-LSCs. *p < 0.05, **p < 0.01.

To interrogate genetic heterogeneity of AML at the stem cell level, we compared mutations and their variant allelic frequencies (VAFs) between bulk leukemic blasts and FACS-purified LSC-enriched and non-LSC-enriched compartments (Fig. 3A). A total of 223 unique pathogenic somatic variants were detected in 88 patients, and 182 of them (82%) were found in all three fractions. There were 11 pathogenic variants found exclusively in LSC-enriched compartment and 12 pathogenic variants were found exclusively in non-LSC-enriched compartment. All 23 variants were subclonal as indicated by their low VAF within these fractions (Fig. 3B). An overview of VAF comparisons for common myeloid mutations between LSCs vs non-LSCs is illustrated in the lollipop plot (Fig. 3C). There were no significant differences for mutations involving DNA methylation pathways (*DNMT3A, TET2, IDH1, IDH2*), spliceosomal machinery (*SRSF2, U2AF1, SF3B1*), as well as *NPM1*, *GATA2* and *WT1* (Fig. 3D). However, mutations involving signaling pathways had lower VAF in LSC compartment as compared to non-LSCs, including *FLT3-*TKD (mean, 12.2% vs 23.7%, *p* = 0.006), *NRAS* (mean, 16.7% vs 25.5%, *p* = 0.002), and *KRAS* (mean, 13.3% vs 18.7%, *p* = 0.05) mutations (Fig. 3E). Last, mutations involving *CEBPA* (mean, 6.8% vs 22.1%, *p* = 0.04) and *TP53* (mean, 66.1% vs 84.5%, *p* = 0.03) showed a similar pattern of enhanced abundance in non-LSCs. There were no significant VAF differences between bulk blast population and non-LSC population for any mutation, which can be explained by the fact that LSCs represent a rare fraction while non-LSCs constitute the bulk of leukemia, and providing further support for our immunophenotypically-defined approach. It has been well-documented that mutations involving signaling pathways are acquired later during clonal progression of AML [22]. Concordantly, our data indicate that mutational composition of LCS-enriched subpopulation shows differences from the blasts constituting the bulk of leukemia, which may be consistent with the sequence of mutations observed during the evolution of AML.

### Preleukemic HSC compartment can be distinguished from LSCs in primary AML samples

**Fig. 4:**
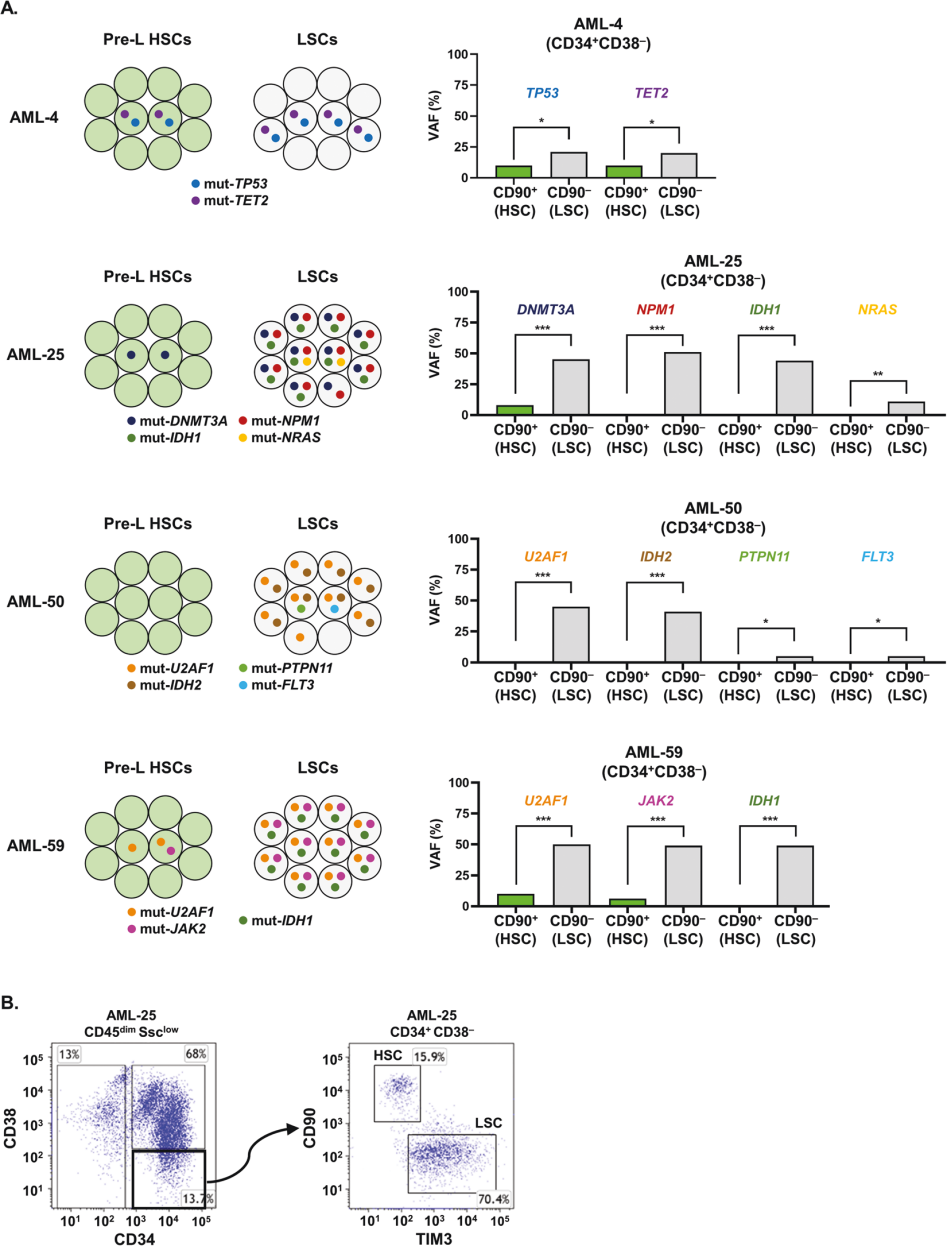
CD90 as a selection marker. **A** Enrichment of immunophenotypically-defined pre-leukemic hematopoietic stem cell (HSC) and leukemia stem cell (LSC) compartments using CD90 as a selection marker. **B** CD90 and TIM3 staining in immunophenotypically-defined stem cell (CD34^+^CD38^‒^) compartment.

Our sorting strategy used CD90 as a marker to separate LSCs from normal or preleukemic HSC compartment, as validated by previous reports [23, 24]. We compared FACS-purified LSC-enriched (CD45^dim^SSc^low^Lin^–^CD90^–^CD34^+^CD38^–^) and preleukemic HSC-enriched (CD45^dim^SSc^low^ Lin^–^CD90^+^CD34^+^CD38^–^) compartments for their mutation profiles in select samples (Fig. 4A). In 4 AML patients with unique mutation compositions, the LSC compartment contained higher number of mutations compared to HSC compartment. The VAF for mutations were also higher in LSCs compared to HSCs and the latter often did not contain the leukemia-associated mutations. In addition, we added TIM3 to the panel to use as an additional LSC-specific marker (Fig. 4B). Within immunophenotypically defined stem cell (CD34^+^CD38^–^) compartment, CD90^+^ and TIM3^+^ cells were separated into two different populations, further confirming the ability of these markers to define these individual populations.

### *TP53*-mutated AML is characterized by stepwise mutation acquisition and clonal selection

**Fig. 5:**
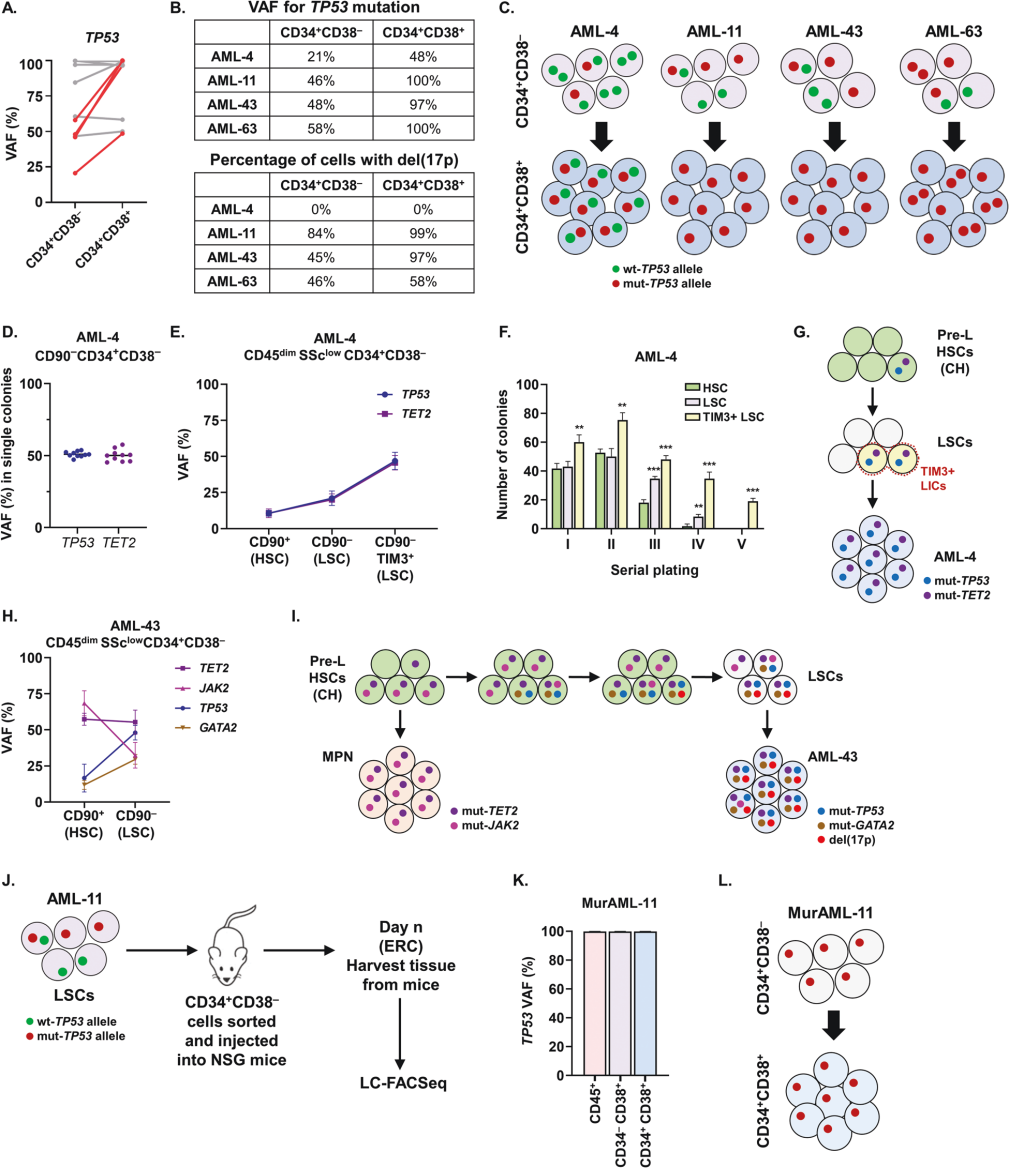
Evolutionary pathways in *TP53* mutated acute myeloid leukemia (AML). **A** Comparison of *TP53* mutation variant allelic frequency (VAF) between immunophenotypic leukemia stem cell (LSC) and non-LSC compartments in 10 AML patients. Gray lines indicate no statistically significant difference, while red lines indicate a significant difference between the two VAFs. **B** Tables show the VAF of *TP53* mutation and percentage of cells with loss of wild type *TP53* allele detected by fluorescence in situ hybridization in 4 AML patients for whom the VAF of mutation doubled from LSC to non-LSC compartment. **C** Schema depicting the evolution of *TP53* mutated AML from LSCs through clonal selection and progression. **D** Graph demonstrates the VAFs for *TP53* and *TET2* mutations in isolated single colonies arising from immunophenotypic LSCs in patient AML-4. **E** Graph demonstrates the VAFs for *TP53* and *TET2* mutations in immunophenotypic hematopoietic stem cell (HSC), LSC and TIM3 + LSCs in patient AML-4. **F** Immunophenotypically defined HSC, LSC and TIM3 + LSCs were isolated from patient AML-4 and cultured in methylcellulose-based MethoCult medium. Graph shows the number of colonies detected at each time point throughout serial replating. **G** Schema depicting the clonal evolution of leukemia in patient AML-4. **H** Graph demonstrates the VAFs for *TET2*, *JAK2*, *TP53*, and *GATA2* mutations in immunophenotypically defined HSCs and LSCs in patient AML-43. **I** Schema depicting the clonal evolution of myeloproliferative neoplasm (MPN) and leukemia in patient AML-43. **J** LSCs from AML-11 were sorted and injected into NSG mice. After meeting early removal criteria (ERC), mice were sacrificed and bone marrow/spleen cells were harvested to perform LC-FACSeq analysis. **K** Graph demonstrates the VAFs for *TP53* mutation in human CD45^+^, CD34^+^CD38^–^, and CD34^+^CD38^+^ compartments of murine AML-11 (murAML-11). **L** Schema depicting the clonal composition of murAML-11. *p < 0.05, **p < 0.01, ***p < 0.001.

Given the significant intercompartmental VAF differences for *TP53* mutation, we wanted to gain insights into the evolution of *TP53*-mutated AML in this cohort. *TP53*-mutated AML is associated with adverse risk cytogenetics, resistance to standard chemotherapy, and poor survival [2]. Among 10 patients with *TP53* mutation in our cohort, 4 patients demonstrated doubling of their mutation VAF across compartments (Fig. 5A). This has led us to hypothesize that self-renewing clones with *TP53* mutation may lose the wild type allele (i.e., loss of heterozygosity [LOH]) as they progress to generate acute leukemia. Thus, we assessed *TP53* gene copy number in LSC-enriched and non-LSC-enriched populations by using FISH probe for 17p13.1 (Supplementary Fig. 2A). Integrating the VAF and copy number information, we inferred the clonal composition in these 4 patients (Fig. 5B, C).

In AML-4, there were no copy number abnormalities, thus 40% of cells in immunophenotypically defined (CD34^+^CD38^–^) LSC compartment harbored one mutated *TP53* allele, while all cells in non-LSC (CD34^+^CD38^+^) compartment were heterozygous for *TP53* mutation, suggesting clonal selection of mutated LSCs. To investigate the colony forming capabilities of genetically heterogenous cells in the LSC-enriched compartment, we FACS-purified CD34^+^CD38^–^ cells and plated in MethoCult medium. After 14 days, we isolated individual colonies and sequenced. All colonies demonstrated heterozygous *TP53* and *TET2* mutations, indicating that only the LSC clones harboring these mutations have the self-renewal capacity to drive leukemia (Fig. 5D). Since these cells represented only 40% of our original marker-defined putative LSCs, we explored additional LSC markers CD99, CD123, and TIM3, to enhance the ability to isolate the most clonogenic cells [25–27]. AML-4 was negative for CD99 and CD123, but we were able to identify a TIM3^+^ LSC subpopulation within the CD34^+^CD38^–^ compartment. Next, we FACS-purified and compared CD34^+^CD38^–^CD90^–^ (LSC), CD34^+^CD38^–^CD90^–^TIM3^+^ (TIM3^+^ LSC), and CD34^+^CD38^–^CD90^+^ (preleukemic HSC) compartments (Fig. 5E). All cells in TIM3^+^ LSC subpopulation were heterozygous for *TET2* and *TP53* mutations, and evidence of these mutations were also found in preleukemic HSCs at a lower VAF. Next, we functionally validated the clonogenic potential of these stem cell compartments by serial plating colony formation assay (Fig. 5F). TIM3^+^ LSCs, which were heterozygous for the mutations, had higher self-renewal capacity than both CD34^+^CD38^–^CD90^–^ (putative LSC) and CD34^+^CD38^–^CD90^+^ (HSC) cells. Combining the genomic and functional characterization of these different fractions, we modeled the leukemogenesis in this patient, originating from preleukemic CH-containing *TET2*/*TP53* mutant HSCs, which give rise to TIM3^+^ LSCs with heightened self-renewal capacity, driving AML with heterozygous *TET2*/*TP53* mutation status (Fig. 5G).

In AML-11, AML-43, and AML-63, *TP53*-mutated AML was characterized by LOH, which was driven by the subfraction of leukemia initiating cells harboring both the mutation and del(17p) within a genetically heterogenous LSC pool. We examined AML-43 in detail, which was a case evolving from an antecedent *JAK2* + *TET2* + myeloproliferative neoplasm (MPN). Preleukemic HSCs and LSCs from this patient were isolated and sequenced (Fig. 5H). This case did not have an LSC subpopulation positively staining with an additional marker (e.g., CD99, CD123, or TIM3), hence we could not subfractionate this compartment. A small fraction of the HSCs harbored *TP53* (VAF 16.6%) and *GATA2* (VAF 11.8%) mutations. FISH in the HSC fraction revealed that 19% of cells were missing one 17p13.1 signal. Taken together, we modeled the development of AML from antecedent CH and MPN in this patient, whereby *TET2*/*JAK2* mutant preleukemic HSCs acquired additional mutations in *TP53* and *GATA2* genes, followed by LOH in a subfraction of LSCs, which had the clonogenic advantage to capitulate AML (Fig. 5I).

To understand the leukemogenic potential of genetically heterogenous cells residing in the LSC compartment, we sorted putative LSCs (CD34^+^CD38^–^) from AML-11 and injected 10,000 cells into NSG mouse (Fig. 5J). After 6 months, the mice developed signs of disease, bone marrow and spleen cells were isolated and designated as murine AML-11 (murAML-11) leukemic cells. Using LC-FACSeq, we purified and sequenced CD45^+^, CD34^+^CD38^–^ and CD34^+^CD38^+^ compartments. Within all compartments, we detected the *TP53* R273H mutation at VAF > 99%, indicating that the LSCs harboring mutation and del(17p) (i.e., loss-of-heterozygosity) had a competitive engraftment advantage over LSCs with del(17p) alone and LSCs containing *TP53* R273H without loss-of heterozygosity (Fig. 5K, L). These data provide evidence that the genetically heterogenous cells residing in the LSC compartment have variable leukemogenic potential, which may be dictated by their molecular composition.

### LC-FACSeq can be used to monitor clonal evolution in rare subpopulations of cells in AML patients

**Fig. 6:**
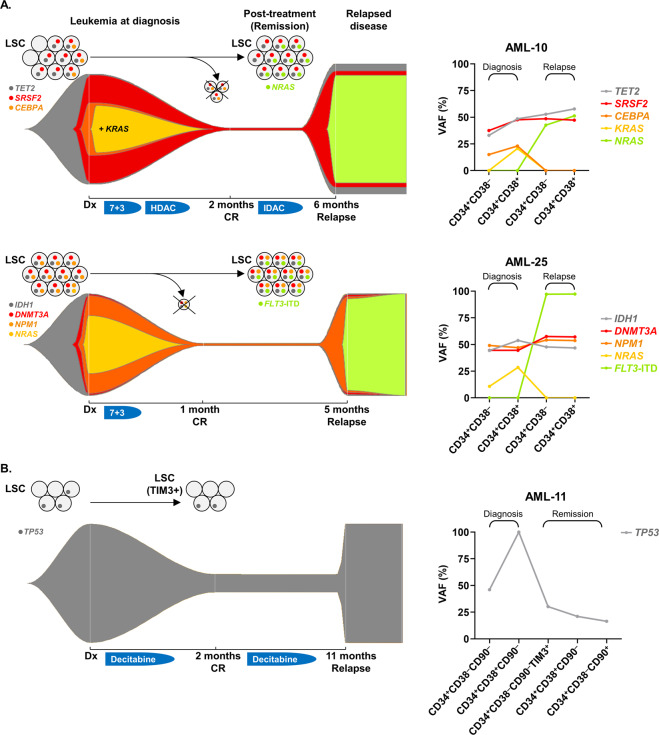
LC-FACSeq can track mutations in leukemia stem cell (LSC) and non-LSC compartments of AML patients at diagnosis, remission and relapse. **A** Fish plots represent the clonal evolution of AML that can be inferred from the variant allelic frequencies (VAF) of somatic mutations detected with LC-FACSeq, which are shown in the corresponding graphs of paired diagnosis and relapse samples. The mutational composition of LSC compartment is also illustrated. **B** Monitorization of *TP53* mutation in patient AML-11 at serial diagnosis, remission and relapse samples. Dx diagnosis, CR complete remission, HDAC high-dose cytarabine, IDAC intermediate-dose cytarabine; 7 + 3 (7-days of cytarabine and 3-days of daunorubicin).

Monitoring MRD and clonal progression throughout the course of disease is essential for therapeutic planning at different time points while on therapy, and after attainment of remission and at relapse. We studied serial diagnosis, remission and relapse samples in patients to track clones with and without high self-renewal potential through lines of treatment. For example, AML-10 contained *TET2* and *SRSF2* mutations, as well as subclonal *CEBPA* mutation in the LSC-enriched compartment at diagnosis (Fig. 6A). The leukemia arising from these LSCs also contained *KRAS* mutation, which was a later event not present in the self-renewing compartment. This patient received intensive induction chemotherapy, which was effective in eradicating LSC subclones with *CEBPA* mutation, but the original *TET2*/*SRSF2* clone persisted when the patient achieved morphologic CR. After consolidation chemotherapy, the *TET2*/*SRSF2* LSC clone acquired *NRAS* mutation, which represented the dominant clone fueling the relapsed disease. Another example, AML-25, had dominant *DNMT3A, NPM1, IDH1* mutations and subclonal *NRAS* mutation at diagnosis. After induction chemotherapy, clones containing *NRAS* mutation were eliminated and the patient achieved morphologic CR. However, other LSC clones persisted and *FLT3*-ITD mutated LSCs were detected at relapse. Other examples for the utility of LC-FACSeq in tracking clonal evolution of AML from diagnosis to relapse can be found in Supplementary Fig. 3A.

AML-11 had complex karyotype and *TP53* mutation at diagnosis, was treated with decitabine and achieved morphologic CR at 2 months post-diagnosis (Fig. 6B). Analysis of this remission sample showed persistent *TP53* mutated LSCs in the TIM3^+^ subfraction, and the patient relapsed after 11 months of decitabine therapy. We had serial diagnosis and remission samples from six additional patients (AML-8, AML-81, AML-82, AML-83, AML-84, AML-85). Crucially, none of them had detectable LSCs or mutations at remission, and they did not experience relapse (Supplementary Fig. 3B).

## Discussion

It has been postulated that AML cells have a hierarchic organization, and LSCs are considered to be the origin of leukemogenesis, treatment failure, and relapse. Our results indicate that the clonal heterogeneity of cells within an individual AML patient may originate from a genetically heterogenous pool of LSCs. LSCs that persist after leukemia therapy provide a reservoir for additional mutations and subsequent relapse. In some cases, relapse may also emerge from a more primitive HSC with CH mutations that re-acquires additional transforming genetic alterations. In addition, LSC frequency at diagnosis was not an independent predictor of patient survival, which suggests that the quantity of these cells at time of diagnosis does not have a direct correlation with their leukemogenic potential. However, LSC population expands as the disease progresses and the LSC frequency at relapse is significantly higher than the pre-treatment frequency. Therefore, frequent monitorization of the mutations and cell frequency in LSC compartment may inform about impending relapse.

We propose LC-FACSeq as a strategy coupling progenitor identification with rigorous cell sorting and targeted deep sequencing, which can empower us to monitor precursor preleukemic conditions such as CH, response to treatment (MRD detection), and clonal evolution at relapse. The technique has been validated in multiple leukemias [20], and the present study exemplifies its role in detecting rare cells throughout disease course. We also examined the clonal compositions of immunophenotypically and functionally defined subpopulations in AML, and provided models for the leukemogenic pathways originating from preleukemic HSCs and CH.

Identification of the genomic landscape of AML enabled the development of several novel molecularly targeted therapies, which entered clinical practice during the past four years and promise to revolutionize the management of this fatal disease. Cure will depend on close monitoring of the disease status and accurate identification and characterization of leukemic cells that resist treatment. Our data and previous studies indicate that LSC burden correlates with poor outcomes [11, 12, 28, 29]. Immunophenotypic identification of LSCs using validated surface markers is faster, easier and more readily clinically available than LSC gene expression scores [20]. We showed that CD34^+^CD38^–^ cells represent a compartment that is enriched with LSCs, and additional markers such as CD123, CD99, or TIM3 may increase the ability to precisely identify cells with most clonogenic potential. However, expression of these additional markers has significant interpatient variability, so clinical trials implementing LSC-focused flow-based MRD detection often utilize an LSC detection tube containing a cocktail of antibodies against these and other markers to increase detection capacity [30]. Flow-based LSC identification as MRD is used now for decisions related to transitioning to allogeneic HCT. However, a gap exists between detection of these cells and exploiting their vulnerabilities to specifically target them with therapeutic agents. LC-FACSeq can be utilized in this setting, as well as in post-HCT remission marrows where novel targeted therapies may be applied to improve outcomes. Bulk sequencing techniques in this setting are unlikely to allow this, even with error correction methods. We are currently improving the sensitivity of LC-FACSeq to develop it as an MRD detection method and compare with traditional MRD methods using bulk leukemia cells as sample source.

We found that AML progenitor compartments contained mutations involving DNA methylation pathways and splicesomal machinery, as well as transcription factors; while signaling pathway mutations were more abundant in non-LSC enriched blasts. LSC clones present at diagnosis were detectable in remission samples of patients who achieved morphologic CR, and these patients later relapsed with disease originating from these clones that might have acquired additional mutations. Importantly, six patients who did not have detectable LSC clones at remission enjoyed long term disease control without relapse. These findings provide a rationale to examine mutations in fractionated LSC population to identify actionable targets to prevent disease progression, which could become a new standard of care as our therapeutic armamentarium continues to expand in AML.

Our study provides a number of key insights into the leukemogenic process in *TP53*-mutated AML. We demonstrated evidence of preleukemic HSCs (CH) with *TP53* mutation in diagnostic samples, which were ancestral to the dominant AML clones. These cells acquired additional mutations in other genes, as well as del(17p) (LOH) to constitute genetic and functional heterogeneity within the LSC pool. Different subclones within the LSC-enriched compartment have varying degrees of clonogenic (leukemia-initiating) potential. Leukemic blasts arising from the LSC pool has a lower degree of genetic heterogeneity and are driven by the LSC subclones with higher self-renewal capacity conferred by accumulation of multiple genetic changes. This sequential stepwise acquisition of mutations can be successfully monitored with LC-FACSeq. These findings have implications for currently employed bulk-cell focused clinical sequencing approaches, which may not have the sensitivity to monitor clonal architecture within a rare population of LSCs arising from CH. Recently, implementation of single cell DNA sequencing techniques in AML provided novel insights into disease pathogenesis by examining clonal architecture at single cell level [31, 32]. However the sensitivity of these techniques in identifying and monitoring mutations in small subpopulations of cells, such as LSCs, needs to be established. Finally, CH with *TP53* mutation is often associated with prior genotoxic therapy and carries a much higher risk for transformation into a myeloid malignancy as compared to more common DTA mutations [18]. Therefore, meticulous monitoring of HSCs and LSCs in this patient population can improve our understanding about the tempo of mutation acquisition and latency to AML development, which can ultimately enable timely preventative therapeutic interventions.

In summary, we interrogated the complex clonal architecture of AML from a stem cell perspective and studied tiny populations of precursor clones, LSCs and MRD with the highly sensitive LC-FACSeq method. Targeting mutations that are concentrated in preleukemic HSCs and LSCs may re-shape the clonal hierarchy and impact on disease course.

## Supplementary information

Supplementary Material

Supplementary Table 1. AML amplicons
