# Correction: Genomic analysis of cellular hierarchy in acute myeloid leukemia using ultrasensitive LC-FACSeq

**DOI:** 10.1038/s41375-021-01398-9

**Published:** 2021-09-03

**Authors:** Caner Saygin, Eileen Hu, Pu Zhang, Steven Sher, Arletta Lozanski, Tzyy-Jye Doong, Deedra Nicolet, Shelley Orwick, Jadwiga Labanowska, Jordan N. Skinner, Casey Cempre, Tierney Kauffman, Virginia M. Goettl, Nyla A. Heerema, Lynne Abruzzo, Cecelia Miller, Rosa Lapalombella, Gregory Behbehani, Alice S. Mims, Karilyn Larkin, Nicole Grieselhuber, Alison Walker, Bhavana Bhatnagar, Clara D. Bloomfield, John C. Byrd, Gerard Lozanski, James S. Blachly

**Affiliations:** 1 Department of Internal Medicine, The Ohio State University, Columbus, OH USA; 2 Division of Hematology, The Ohio State University Comprehensive Cancer Center, Columbus, OH USA; 3 Alliance Statistics and Data Center, The Ohio State University, Columbus, OH USA; 4 Department of Pathology, The Ohio State University, Columbus, OH USA

**Keywords:** Acute myeloid leukaemia, Cancer genetics

Correction to: *Leukemia*; 10.1038/s41375-021-01295-1

The article Genomic analysis of cellular hierarchy in acute myeloid leukemia using ultrasensitive LC-FACSeq, written by Caner Saygin, Eileen Hu, Pu Zhang, Steven Sher, Arletta Lozanski, Tzyy-Jye Doong, Deedra Nicolet, Shelley Orwick, Jadwiga Labanowska, Jordan N. Skinner, Casey Cempre, Tierney Kauffman, Virginia M. Goettl, Nyla A. Heerema, Lynne Abruzzo, Cecelia Miller, Rosa Lapalombella, Gregory Behbehani, Alice S. Mims, Karilyn Larkin, Nicole Grieselhuber, Alison Walker, Bhavana Bhatnagar, Clara D. Bloomfield, John C. Byrd, Gerard Lozanski & James S. Blachly, was originally published electronically on the publisher’s internet portal on 21 May 2021 without open access. With the author(s)’ decision to opt for Open Choice the copyright of the article changed on 16 August 2021 to © The Author(s) 2021 and the article is forthwith distributed under a Creative Commons Attribution.

