# Supplementary material for: Genomic analysis of cellular hierarchy in acute myeloid leukemia using ultrasensitive LC-FACSeq
Source: Leukemia. 2021 May 21;35(12):3406–20. doi: 10.1038/s41375-021-01295-1 (PMC8606012; doi:10.1038/s41375-021-01295-1)
Supplement: Supplementary file 1 — Supplementary Material [file 41375_2021_1295_MOESM1_ESM.docx]

**SUPPLEMENTARY FIGURES**


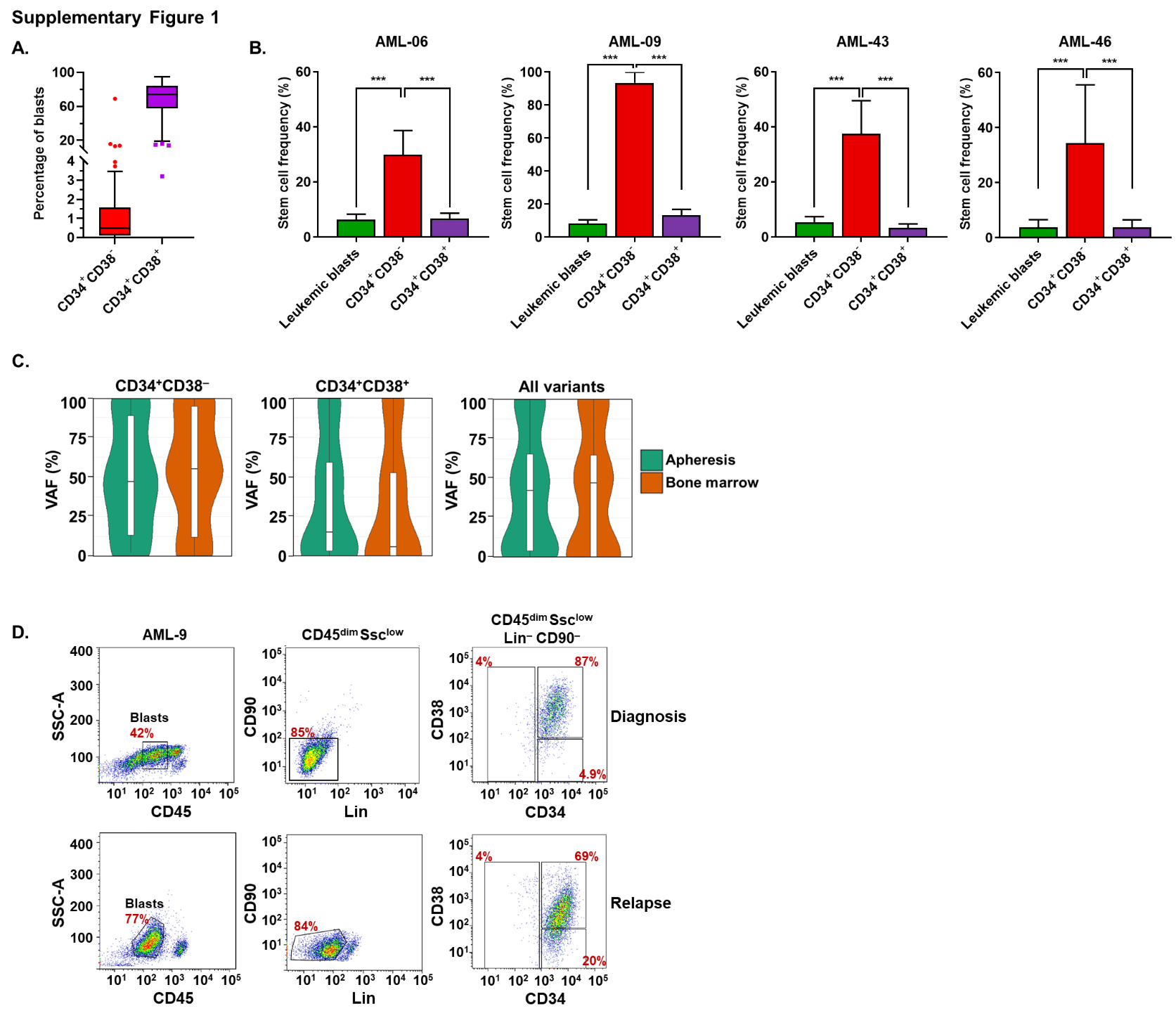


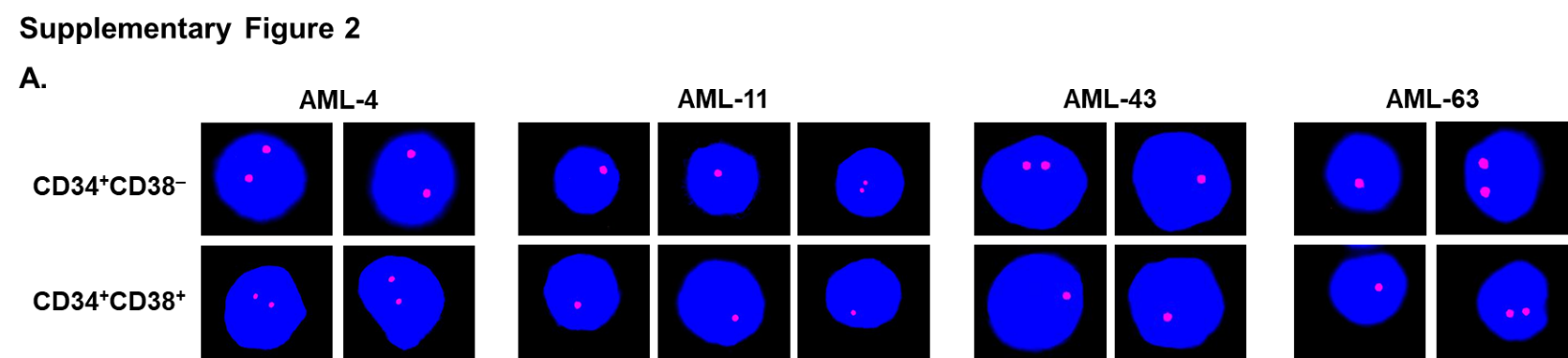


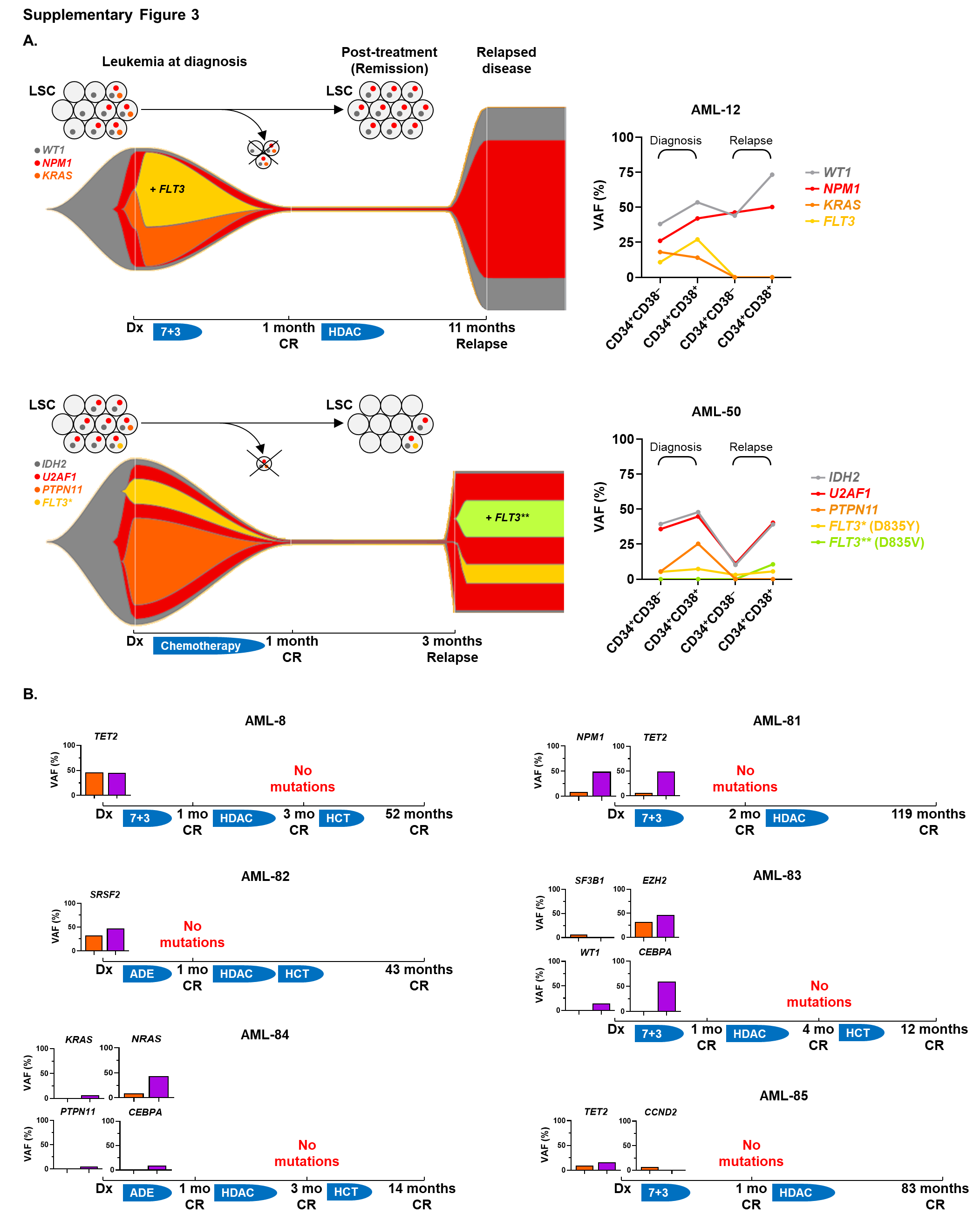


**SUPPLEMENTARY FIGURE LEGENDS**

**Supplementary Figure 1. (A)** Bar graph shows the frequency of immunophenotypically-defined LSC and non-LSC compartments in 88 AML patients. **(B)** In vitro limiting dilution analysis of sorted populations of leukemic blasts, LSCs and non-LSCs in different AML patient samples. Bar graphs represents the estimates in percentage of self-renewal frequency. **(C)** Violin plots comparing the variant allelic frequencies (VAF) of variants detected in different compartments between different sampling sites. **(D)** Flow plots demonstrate the frequencies of LSC and non-LSC compartments in patient AML-9 at the time of diagnosis and relapse. *p< .05, **p< .01, ***p< .001

**Supplementary Figure 2. (A)** Representative fluorescence in-situ hybridization images for immunophenotypic LSCs (CD45^dim^SSc^low^Lin^–^CD90^–^CD34^+^CD38^–^) and non-LSCs (CD45^dim^SSc^low^Lin^–^CD90^–^CD34^+^CD38^+^) of primary AML patient cells probed for *TP53* gene locus at 17p (red).

**Supplementary Figure 3. (A)** Fish plots represent the clonal evolution of AML that can be inferred from the variant allelic frequencies (VAF) of somatic mutations detected with LC-FACSeq, which are shown in the corresponding graphs of paired diagnosis and relapse samples. The mutational composition of LSC compartment is also illustrated. (**B)** Clinical course of 6 patients for whom remission samples did not contain any mutations in either compartment. ADE, adriamycin + doxorubicin + etoposide; CR, complete remission; Dx, diagnosis; HDAC, high-dose cytarabine; 7+3 (7-days of cytarabine and 3-days of daunorubicin)

**SUPPLEMENTARY TABLE LEGENDS**

**Supplementary Table 1.** Amplicon sequences used in LC-FACSeq panel (see excel file in supplementary appendix).
